# 3-D electromagnetic radiative non-Newtonian nanofluid flow with Joule heating and higher-order reactions in porous materials

**DOI:** 10.1038/s41598-020-71543-4

**Published:** 2020-09-03

**Authors:** Amel A. Alaidrous, Mohamed R. Eid

**Affiliations:** 1 Department of Mathematics, Faculty of Applied Sciences, Umm Al-Qura University, Makkah, 21955 Saudi Arabia; 2 Department of Mathematics, Faculty of Science, New Valley University, Al-Kharga, Al-Wadi Al-Gadid, 72511 Egypt; 3 Department of Mathematics, Faculty of Science, Northern Border University, Arar, 1321 Saudi Arabia

**Keywords:** Applied mathematics, Computational science

## Abstract

The aim of this work is to discuss the effect of *m*th-order reactions on the magnetic flow of hyperbolic tangent nanofluid through extending surface in a porous material with thermal radiation, several slips, Joule heating, and viscous dissipation. In order to convert non-linear partial differential governing equations into ordinary ones, a technique of similarity transformations has been implemented and then solved using the OHAM (optimal homotopy analytical method). The outcomes of novel effective parameters on the non-dimensional interesting physical quantities are established utilizing the tabular and pictorial outlines. After a comparison with previous literature studies, the results were finely compliant. The study explores that the reduced Nusselt number is diminished for the escalating values of radiation, porosity, and source (sink) parameters. It is found that the order of the chemical reaction *m* = 2 is dominant in concentration as well as mass transfer in both destructive and generative reactions. When *m* reinforces for a destructive reaction, mass transfer is reduced with 34.7% and is stabled after *η* = 3. In the being of the destructive reaction and Joule heating, the nanofluid's temperature is enhanced.

## Introduction

The heat movement for a viscous fluid has many manufacturing, bio-medical and engineering applications like a capacity generator, rock-oil productions, plasma research, cancer treatment, aerodynamics laminar boundary-layer predominance, and many more. But for that place, this idea takes a long journey to shape up. In 1904, Prandtl proposed the idea of laminar boundary-layers that the viscous effect should be confined to thin shear surfaces adjacent to boundaries in the situation of very low-viscosity fluids^1^. Sakiadis^2,3^ probed the boundary layer Blasius movement wing to the surface being supplied at relaxation with constant velocity from a slit into a stream. Crane^4^ examined the movement over an extending plate. Many academics for example^5,6^ extended the Crane^4^ work. Various researchers are elaborated on various features of these problems for example^7–10^. Different numerical investigations of heat transfer under various effects of one-phase nanofluids flow are elaborated in^11–14^. Gholinia et al.^15^ analyzed the characterization of the 3-D stagnation point hybrid nanofluid flowing over a circulatory cylinder with sinusoidal length. Numerical aspects of 3-D free convection MHD GO-MoS_2_/H_2_O-C_2_H_6_O_2_ mixture nanofluid with radiation and slip effects is are debated by Ghadikolaei and Gholinia^16^. Whilst the flow of free convection past a surface in a porous medium is checked by Hady et al.^17,18^ of a nanofluid through a cone. They achieved solutions by utilizing MATLAB techniques such as bvp4c or Runge–Kutta method.

In chemistry, manufacturing, energy storage, biomedical application, drug delivery based on temperature-controlled, energy storage and conversion, nanoparticles impact plays a very significant role^19^. Ray is used in human forms for drugs, mining, hospital radiology, MRI scans, etc. Heat transfer properties of viscous fluid flow with radiation and nanoparticles via a non-linearly extending surface are elucidated by Hady et al.^20^. Kumar et al.^21^ described the MHD and chemically reacting effects of 3-D non-Newtonian fluid flow past porous material. Hamid et al.^22^ analyzed the flow of free convection non-Newtonian Casson fluid in a trapezoidal cavity. Other important studies in the flow of non-Newtonian fluid are found in^23,24^. Combined impacts of MHD solar radiation, Joule heating, chemical reaction, and viscous dissipation, on the nanofluid flow past a heated expanding plate immersed in a saturated porous matrix, are demonstrated by Eid and Makinde^25^. Eid et al.^26^ analysis of magnetic flow through a circular cylinder of SWCNTs based-blood in with non-linear radiation and source (sink) of heat.

In construction, electronics and heat transport process in the emerging biotechnology and nanotechnology manufacture, etc., MHD and chemical reactions play an important role. In MHD there are sundry applications which are astronomy, earthquakes, metal adaptors, etc. In an exponentially expanding surface, Eid^27^ clarified the issue of the chemically reactive species on and heat source (sink) influences on MHD nanofluid flow via a porous surface. Whilst, the combined impacts of hydro-magnetic and heat source (sink) on unstable convective heat and mass transfer of a power-law nanofluid through a permeable stretch plate are addressed by Eid and Mahny^28^. Durgaprasad et al.^29^ Hassan analyzed the 3-D Casson nanofluid flow using the Buongiorno model across a slender surface of a porous regime. Prasad et al.^30^ screened over a semi-infinite plate with an analytical approximation on MHD flow of nanofluid. Time-dependent Couette flow with heat transfer under the combined effects of thermal radiation and MHD with varying thermo-physical properties are explored numerically for Cu–H_2_O nanofluids by Wakif et al.^31^. Over an extending plate with the influences of heat and mass transport on hydro-magnetic nanofluid flow are deliberated by Gholinia et al.^32^. In this article, they utilized Runge–Kutta scheme to offer graphical exemplification. Heat transfer of a squeezed time-dependent Fe_3_O_4_–H_2_O nanofluid movement among two-parallel surfaces in case of variable thermal conductivity is explored by Lahmar et al.^33^ in the influence of the magnetic field.

The irregular tendency of nanofluid in modern days is due to its unforeseen thermal characteristics and has a dynamic part in enhancing the heat transport of material transmutation and manufacturing thermal management. The discussion of the heat transfer features of gold nanoparticles past a power-law extending plate of Sisko flow based-blood with radiation is elaborated by Eid et al.^34^. The impacts of slip and heat source (sink) on the unsteady heat transfer of a stagnation point nanofluid flow via an extending plate in a porous substance are determined by Eid^35^. The Marangoni influences on the nanofluid flowing of Casson type in with convective conditions are described by Kumar et al.^36^. Several investigators intensively reviewed the transfer of heat of nanofluids such as^37–45^. A variety of scholars have already documented a useful study of the hyperbolic tangent fluid model that preserves various flow phenomena. MHD of convectively heated and condensed tangent hyperbolic nanofluid over an extending surface on the slip flux is showed by Ibrahim^46^. Over an extended surface with the radiative flow of convective tangent hyperbolic liquid is illustrated by Mahanthesh et al.^47^. The issue of tangent-hyperbolic fluid flow past various types of shapes with different impacts of parameters attracted to the attention of many researchers like^48–52^.

Examination of these studies shows that incompressible 3-D tangent hyperbolic nanofluid flowing across a permeable surface with higher-order reactions are not studied. It is accounted for the combined effect of heating the Joule heating and viscous dissipation in a porous medium with the effects of the heat source (sink), slip velocity, and thermal radiation. The governing equations of the problem are solved by converting PDEs into ODEs by utilizing similarity transformations. OHAM was also used for related similar solutions. The findings were presented by figures and tables for the different parameters. Numerical computations are determined and presented for the drag force, local Nusselt, and Sherwood numbers.

## Problem analysis

**Figure 1:**
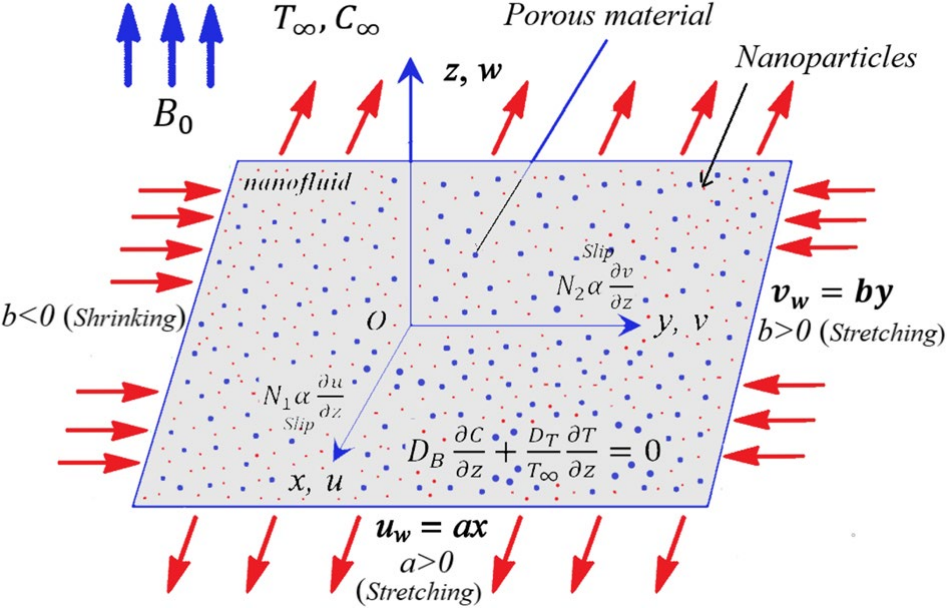
Geometric flow model.

Consider the steady, incompressible non-Newtonian hyperbolic tangent nanofluid flow through a porous extending surface (see Fig. 1) with the assumptions:

- The flow model is 3-D with the *z*-direction perpendicular to the surface which is extended in the *xy*-plane.
- The velocity of mass influx is $w = w_{0}$, where $w_{0} < 0$ mentioned for injection, and $w_{0} > 0$ is for suction.
- Joule heating, MHD flow, higher-order chemical reaction, and viscous dissipation are considered in a porous medium.

The boundary-layer equations system of mass, motion, heat, and volume concentration for the flow under the above aspects according to Buongiorno model are^37,41,49,51^:

1 $$ \frac{\partial u}{{\partial x}} + \frac{\partial v}{{\partial y}} + \frac{\partial w}{{\partial z}} = 0, $$

2 $$ u\frac{\partial u}{{\partial x}} + v\frac{\partial u}{{\partial y}} + w\frac{\partial u}{{\partial z}} = \nu (1 - n)\frac{{\partial^{2} u}}{{\partial z^{2} }} + n\nu \sqrt 2 \Gamma \frac{\partial u}{{\partial z}}\frac{{\partial^{2} u}}{{\partial z^{2} }} - \frac{{\sigma B_{0}^{2} }}{\rho }u - \frac{\nu }{{K_{1} }}u, $$

3 $$ u\frac{\partial v}{{\partial x}} + v\frac{\partial v}{{\partial y}} + w\frac{\partial v}{{\partial z}} = \nu (1 - n)\frac{{\partial^{2} v}}{{\partial z^{2} }} + n\nu \sqrt 2 \Gamma \frac{\partial v}{{\partial z}}\frac{{\partial^{2} v}}{{\partial z^{2} }} - \frac{{\sigma B_{0}^{2} }}{\rho }v - \frac{\nu }{{K_{1} }}v $$

4 $$ u\frac{\partial T}{{\partial x}} + v\frac{\partial T}{{\partial y}} + w\frac{\partial T}{{\partial z}} = \alpha \frac{{\partial^{2} T}}{{\partial z^{2} }} + \tau \left[ {D_{B} \frac{\partial C}{{\partial z}}\frac{\partial T}{{\partial z}} + \frac{{D_{T} }}{{T_{\infty } }}\left( {\frac{\partial T}{{\partial z}}} \right)^{2} } \right] + \frac{{\sigma B_{0}^{2} }}{{\rho c_{p} }}u^{2} + \frac{Q}{{\rho c_{p} }}(T - T_{\infty } ) - \frac{1}{{\rho c_{p} }}\frac{{\partial q_{r} }}{\partial z} + \frac{\nu }{{c_{p} }}(1 - n)\left( {\frac{\partial u}{{\partial z}}} \right)^{2} , $$

5 $$ u\frac{\partial C}{{\partial x}} + v\frac{\partial C}{{\partial y}} + w\frac{\partial C}{{\partial z}} = D_{B} \frac{{\partial^{2} C}}{{\partial z^{2} }} + \frac{{D_{T} }}{{T_{\infty } }}\frac{{\partial^{2} T}}{{\partial z^{2} }} - k_{c} (C - C_{\infty } )^{m} . $$

The related boundary conditions are

6 $$ \left. {\begin{array}{*{20}l} {u = u_{w} (x) = ax + N_{1} \alpha \frac{\partial u}{{\partial z}}, v = v_{w} (y) = by + N_{2} \alpha \frac{\partial v}{{\partial z}},} \\ {w = w_{0} , \;T = T_{w} ,\; D_{B} \frac{\partial C}{{\partial z}} + \frac{{D_{T} }}{{T_{\infty } }}\frac{\partial T}{{\partial z}} = 0 \;\;{\text{at}}\;\; z = 0, } \\ {u \to 0, \;v \to 0, \;T \to T_{\infty } , \;C \to C_{\infty } \;\;{\text{as}}\;\; z \to \infty , } \\ \end{array} } \right\} $$

where *u*, *v*, and *w* are the velocity coordinates of along the orientations of *x*-, *y*-, and *z*-coordinates, respectively. Further, suppose that $$u_{w} (x) = ax$$ and $$v_{w} (y) = by$$, anywhere $$a > 0$$, and $$b$$ is a constant, when $$(b > 0) $$ agrees with an extending surface and whilst $$(b < 0)$$ matches to a shrinking surface. The condition $$D_{B} \frac{\partial C}{{\partial z}} + \frac{{D_{T} }}{{T_{\infty } }}\frac{\partial T}{{\partial z}} = 0$$ at $$z = 0$$ physically implies that the nanoparticle fraction at the wall is measured passively. This condition provides for a random motion of nanoparticles at the boundary-layer. According to the Rosseland approximation, $$q_{r}$$ is calculated as:

7 $$ q_{r} = - \frac{{4\sigma^{*} }}{{3k^{*} }}\frac{{\partial T^{4} }}{\partial z}. $$

Supposing that temperature variance within the flux is such that $T^{4}$ can be prolonged in a Taylor-series about $T_{\infty }$ and disregarding higher-order relationships. This outcome is the subsequent approximation: $T^{4} \cong 4T_{\infty }^{3} T - 3T_{\infty }^{4}$. Then, the relation (7) can be rewritten as $\frac{{\partial q_{r} }}{\partial z} = - \frac{{16\sigma^{*} T_{\infty }^{3} }}{{3k^{*} }}\frac{{\partial^{2} T}}{{\partial z^{2} }}$ and moreover the heat relation (4) becomes in the formula:

8 $$ u\frac{\partial T}{{\partial x}} + v\frac{\partial T}{{\partial y}} + w\frac{\partial T}{{\partial z}} = \left( {\alpha + \frac{1}{{\rho c_{p} }}\frac{{16\sigma^{*} T_{\infty }^{3} }}{{3k^{*} }}} \right)\frac{{\partial^{2} T}}{{\partial z^{2} }} + \tau \left[ {D_{B} \frac{\partial C}{{\partial z}}\frac{\partial T}{{\partial z}} + \frac{{D_{T} }}{{T_{\infty } }}\left( {\frac{\partial T}{{\partial z}}} \right)^{2} } \right] + \frac{{\sigma B_{0}^{2} }}{{\rho c_{p} }}u^{2} + \frac{Q}{{\rho c_{p} }}(T - T_{\infty } ) + \frac{\nu }{{c_{p} }}(1 - n)\left( {\frac{\partial u}{{\partial z}}} \right)^{2} . $$

### Similarity transformations

Applying the next similarity transformations to the equations of the model and the associated boundary-conditions^51^,

9 $$ \left. {\begin{array}{*{20}l} {u = axf^{\prime}(\eta ), \;v = ay{\text{g}}^{\prime}(\eta ),\; w = - \sqrt {a\alpha } \left[ {f(\eta ) + g(\eta )} \right],\; \eta = \sqrt {\frac{a}{\alpha }} z} \\ {\theta (\eta ) = \frac{{T - T_{\infty } }}{{T_{w} - T_{\infty } }}, \;\phi (\eta ) = \frac{{C - C_{\infty } }}{{C_{w} - C_{\infty } }}, } \\ \end{array} } \right\} $$

where the primes (′) signify the differentiation with respect to $\eta$.

The flow model Eqs. (1–6) becomes:

10 $$ \left[ {(1 - n) + nWe_{x} f^{\prime\prime}} \right]Pr\;f^{\prime\prime\prime} - f^{{\prime}{2}} + (f + {\text{g}})f^{\prime\prime} - Mf^{\prime} - Kf^{\prime} = 0, $$

11 $$ (f + g)g^{\prime\prime} - g^{{\prime}{2}} + \left[ {(1 - n) + nWe_{y} g^{\prime\prime}} \right]Pr\;g^{\prime\prime\prime} - Mg^{\prime} - Kg^{\prime} = 0, $$

12 $$ \left( {1 + \frac{4}{3}R_{d} } \right)\theta^{\prime\prime} + (f + g)\theta^{\prime} + N_{b} \theta^{\prime}\phi^{\prime} + N_{t} \theta^{{\prime}{2}} + MEcf^{{\prime}{2}} + S\theta + (1 - n)\Pr Ecf^{{\prime\prime}{2}} = 0, $$

13 $$ \phi^{\prime\prime} + Le(f + {\text{g}})\phi^{\prime} + \frac{{N_{t} }}{{N_{b} }}\theta^{\prime\prime} - \gamma Le\phi^{m} = 0 $$

and the boundary-conditions in dimensionless forms are

14 $$ \left. {\begin{array}{*{20}l} {f^{\prime}(0) = 1 + \delta_{1} f^{\prime\prime}(0), \;{\text{g}}^{\prime}(0) = \lambda + \delta_{2} {\text{g}}^{\prime\prime}(0),\; f(0) = f_{w} ,} \\ {g(0) = 0, \;\theta (0) = 1, \;\phi^{\prime}(0) + \frac{{N_{t} }}{{N_{b} }}\theta^{\prime}(0) = 0, } \\ {f^{\prime}(\infty ) = 0, \;{\text{g}}^{\prime}(\infty ) = 0, \;\theta (\infty ) = 0, \;\phi (\infty ) = 0, } \\ \end{array} } \right\} $$

where $\lambda$ characterizes the constant parameter of the sheet, where $\lambda > 0$ implies an extending plate whereas $\lambda < 0$ is exposes a shrinking plate. In this investigation, $f_{w}$ represents the suction (injection) parameter, somewhere $f_{w} > 0$ resembles to suction, whilst $f_{w} < 0$ sympathizes to the blowing and $f_{w} = 0$ signifies an impermeable sheet. The values of used parameters are known as follows:

$$ \begin{gathered} Pr = \frac{\nu }{\alpha },\;\;We_{x} = \sqrt {2\frac{a}{\alpha }} a{\Gamma }x,\;\;We_{y} = \sqrt {2\frac{a}{\alpha }} a{\Gamma }y,\;\;M = \frac{{\sigma B_{0}^{2} }}{a\rho },\;\;K = \frac{\nu }{{aK_{1} }},\;\;\delta_{1} = \sqrt {a\alpha } N_{1} ,\;\;\delta_{2} = \sqrt {a\alpha } N_{2} ,\;\;\lambda = \frac{b}{a}, \hfill \\ f_{w} = \frac{{ - w_{0} }}{{\sqrt {a\alpha } }},\;\;Le = \frac{\alpha }{{D_{B} }},\;\;N_{b} = \frac{{\tau D_{B} (C_{w} - C_{\infty } )}}{\alpha },\;\;N_{t} = \frac{{\tau D_{T} (T_{w} - T_{\infty } )}}{{\alpha T_{\infty } }},\;\;R_{d} = \frac{{4\sigma^{*} T_{\infty }^{3} }}{{3k^{*} k}},\;\;Ec = \frac{{u_{w}^{2} }}{{c_{p} (T_{w} - T_{\infty } )}} \hfill \\ S = \frac{Q}{{a\rho c_{p} }},\;\;{\text{and}}\;\;\gamma = \frac{{k_{c} (C_{w} - C_{\infty } )^{m - 1} }}{a}. \hfill \\ \end{gathered} $$

### Drag force, heat and mass transfer

The drag force coefficients $C_{fx}$, $C_{fy}$, the Nusselt $Nu_{x}$ and the Sherwood numbers $Sh_{x}$ are specified as^49^

15 $$ C_{fx} = \frac{{\tau_{wx} }}{{\rho U_{w}^{2} }},\;\;C_{fy} = \frac{{\tau_{wy} }}{{\rho V_{w}^{2} }},\;\;Nu_{x} = \frac{{xq_{w} }}{{k(T_{w} - T_{\infty } )}}\;\;{\text{and}}\;\;Sh_{x} = \frac{{xq_{m} }}{{D_{B} (C_{w} - C_{\infty } )}}, $$

wherever $\tau_{wx}$ and $\tau_{wy}$ are the coefficients of skin friction in the $x$ and $y$ coordinates, $q_{w}$ and $q_{m}$ are the heat and mass fluxes from the wall of the surface. These are given as^49^

16 $$ \left. {\begin{array}{*{20}l} {\tau_{wx} = \mu \left[ {(1 - n)\frac{\partial u}{{\partial z}} + \frac{n\Gamma }{{\sqrt 2 }}\left( {\frac{\partial u}{{\partial z}}} \right)^{2} } \right]_{z = 0} , \;\;\tau_{wy} = \mu \left[ {(1 - n)\frac{\partial v}{{\partial z}} + \frac{n\Gamma }{{\sqrt 2 }}\left( {\frac{\partial v}{{\partial z}}} \right)^{2} } \right]_{z = 0} } \\ {q_{w} = - k\left( {\frac{\partial T}{{\partial z}}} \right)_{z = 0} , \;\;q_{m} = - D_{B} \left( {\frac{\partial C}{{\partial z}}} \right)_{z = 0} ,} \\ \end{array} } \right\} $$

17 $$ \left( {\frac{{Re_{x} }}{Pr}} \right)^{1/2} C_{fx} = \left[ {(1 - n)f^{\prime\prime}(0) + \frac{{nWe_{x} }}{2}f^{{\prime\prime}{2}} (0)} \right], $$

18 $$ \left( {\frac{{Re_{y} }}{Pr}} \right)^{1/2} (\lambda )^{3/2} C_{fy} = \left[ {(1 - n)g^{\prime\prime}(0) + \frac{{nWe_{y} }}{2}g^{{\prime\prime}{2}} (0)} \right] $$

19 $$ Nu_{x} = - \left( {1 + \frac{4}{3}R_{d} } \right)\left( {Pr\;Re_{x} } \right)^{1/2} \theta^{\prime}(0), $$

20 $$ Sh_{x} = - \left( {Pr\;Re_{x} } \right)^{1/2} \phi^{\prime}(0), $$

where $Re_{x} = \frac{{xu_{w} }}{\nu }$ and $Re_{x} = \frac{{yv_{w} }}{\nu }$ are the local Reynolds numbers.

## Homotopic solution

Today, the computational methods used to solve the non-linear equations usually use approximations for non-linear terms or solutions to discretization or linearization. In engineering, challenges occur, and traditional numerical methods cannot always be used to address technology. Researchers have taken a tremendous interest in the analytical solution of these kinds of nonlinear issues over the last few decades. HAM is a type of method of analytical approximation majorly for non-linear differential equations. Observe that HAM utilizes a much more complicated equation of homotopy than for the continuation method of homotopy. In addition, the HAM gives greater freedom to choose the linear auxiliary operator. Most notably, the so-named convergence control parameter is introduced in the homotopy equation for the first time, such that the HAM affords us with an unpretentious way to ensure sequence convergence. The fundamental concept behind the method of analyzing homotopy is given by Liao^53^. He applied a definition of topology renowned as homotopy. He exercised two distinct continuous functions specified by $\zeta_{1} (\tilde{x})$ and $\zeta_{2} (\tilde{x})$ via the two spaces $\hat{X}$ and $\hat{\Upsilon }$. The general structure of the transformation is depending on connecting the closed unit interval with the defined topological spaces, as shown below:

21 $$ \tilde{\Psi }:\hat{X} \times [0,\;1] \to \hat{\Upsilon } $$

where $\tilde{\Psi }[\tilde{x},\;0] = \zeta_{1} (\tilde{x})$ and $\tilde{\Psi }[\tilde{x},\;1] = \zeta_{2} (\tilde{x})$, $\forall \tilde{x} \in \hat{X}$. In Eq. (20), the given process is called a homotopic conversion. The system of ODEs (10)–(13) with the boundary conditions (14) are resolved by OHAM, with selecting appropriate initial estimations $f_{0}$, ${\text{g}}_{0}$, $\theta_{0}$ and $\phi_{0}$ with the consistent linear operators specified as:

22 $$ f_{0} = 1 - e^{ - \eta } ,\; {\text{g}}_{0} = \lambda (1 - e^{ - \eta } ),\; \theta_{0} = e^{ - \eta } , \;\;{\text{and}}\;\; \phi_{0} = \frac{{N_{t} }}{{N_{b} }}e^{ - \eta } $$

23 $$ L_{f} = (D^{3} - D)f,\;\; L_{{\text{g}}} = (D^{3} - D)g, \;\;L_{\theta } = (D^{2} - D)\theta , \;\;{\text{and}}\;\; L_{\phi } = (D^{2} - D)\phi $$

with

24 $$  \left.  \begin{array}{l}{L_{f} =\left( {\sum\nolimits_{i = 0}^{2} {{-}\!\!\!\text{D}_{i + 1} e^{(i - 1)\eta } } } \right) = 0,} \\ {L_{{\text{g}}} = \left( {\sum\nolimits_{i = 0}^{2} {{-}\!\!\!\text{D}_{i + 4} e^{(i - 1)\eta } } } \right) = 0,} \\ {L_{\theta } = \left( {\sum\nolimits_{i = 2}^{3} {{-}\!\!\!\text{D}_{i + 5} e^{{( - 1)^{i} \eta }} } } \right) = 0,} \\ {L_{\phi } = \left( {\sum\nolimits_{i = 2}^{3} {{-}\!\!\!\text{D}_{i + 7} e^{{( - 1)^{i} \eta }} } } \right) = 0,}\end{array} \right\} $$

where ${-}\!\!\!\text{D}_{i} \;(i = 1 - 10)$ signify the constants

**Figure 2:**
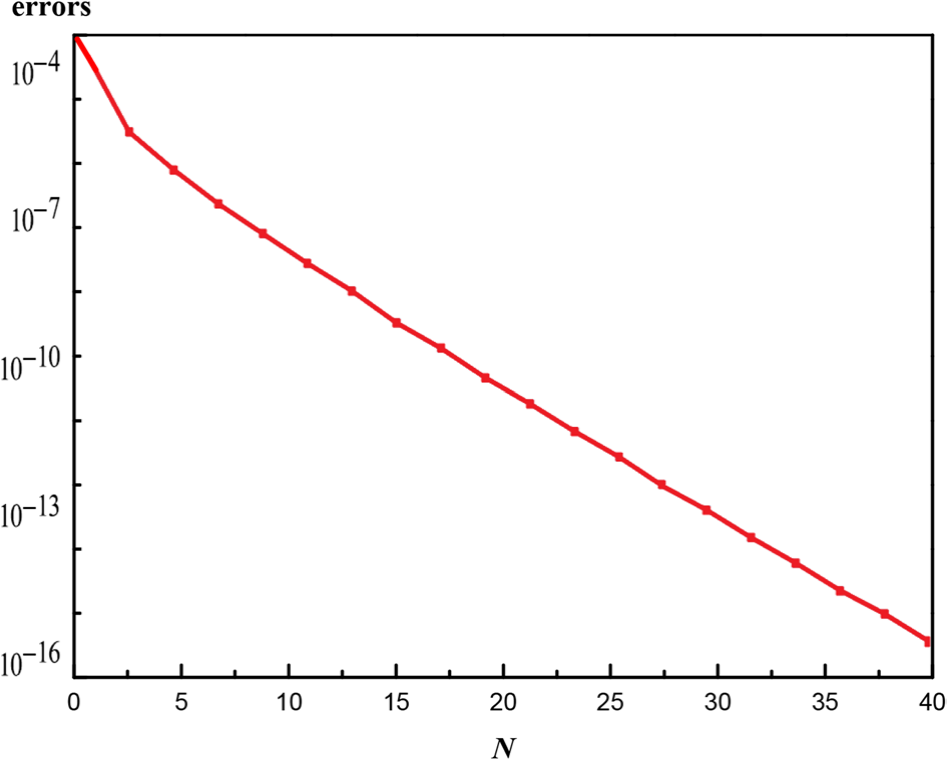
Sum average of residual squared errors.

The errors are appointed in $N{\text{th}}$-order as:

25 $$ e_{N}^{f} = \frac{1}{k + 1}\mathop \sum \limits_{i = 0}^{k} \left[ {N_{f} \left( {\mathop \sum \limits_{j = 0}^{N} \left( {\hat{f}_{j} } \right)_{\eta = i\pi \eta } , \;\;\mathop \sum \limits_{j = 0}^{N} \left( {{\hat{\text{g}}}_{j} } \right)_{\eta = i\pi \eta } } \right)} \right]^{2} $$

26 $$ e_{N}^{{\text{g}}} = \frac{1}{k + 1}\mathop \sum \limits_{i = 0}^{k} \left[ {N_{{\text{g}}} \left( {\mathop \sum \limits_{j = 0}^{N} \left( {\hat{f}_{j} } \right)_{\eta = i\pi \eta } , \mathop \sum \limits_{j = 0}^{N} \left( {{\hat{\text{g}}}_{j} } \right)_{\eta = i\pi \eta } } \right)} \right]^{2} $$

27 $$ e_{N}^{\theta } = \frac{1}{k + 1}\mathop \sum \limits_{i = 0}^{k} \left[ {N_{\theta } \left( {\mathop \sum \limits_{j = 0}^{N} \left( {\hat{f}_{j} } \right)_{\eta = i\pi \eta } , \mathop \sum \limits_{j = 0}^{N} \left( {{\hat{\text{g}}}_{j} } \right)_{\eta = i\pi \eta } , \mathop \sum \limits_{j = 0}^{N} \left( {\hat{\theta }_{j} } \right)_{\eta = i\pi \eta } , \mathop \sum \limits_{j = 0}^{N} \left( {\hat{\phi }_{j} } \right)_{\eta = i\pi \eta } } \right)} \right]^{2} $$

28 $$ e_{N}^{\phi } = \frac{1}{k + 1}\mathop \sum \limits_{i = 0}^{k} \left[ {N_{\phi } \left( {\mathop \sum \limits_{j = 0}^{N} \left( {\hat{f}_{j} } \right)_{\eta = i\pi \eta } , \mathop \sum \limits_{j = 0}^{N} \left( {{\hat{\text{g}}}_{j} } \right)_{\eta = i\pi \eta } , \mathop \sum \limits_{j = 0}^{N} \left( {\hat{\theta }_{j} } \right)_{\eta = i\pi \eta } , \mathop \sum \limits_{j = 0}^{N} \left( {\hat{\phi }_{j} } \right)_{\eta = i\pi \eta } } \right)} \right]^{2} $$

29 $$ e_{N}^{t} = e_{N}^{f} + e_{N}^{{\text{g}}} + e_{N}^{\theta } + e_{N}^{\phi } $$

where $$e_{N}^{t}$$ describes the sum of square remaining error. The main purpose is to accomplish optimum converged parameters by reducing the average square residues in total. The variable $$\hbar$$ is called the convergence control plays a crucial part. At $$4{\text{th}}$$-order of computations, the squared residual errors $$\hbar_{f}$$, $$\hbar_{{\text{g}}}$$$$\hbar_{\theta }$$, $$\hbar_{\phi }$$ are $$\hbar_{f} = - 1.499782$$, $$\hbar_{{\text{g}}} = - 1.070914$$,$$ \hbar_{\theta } = - 0.849217$$ and $$ \hbar_{\phi } = - 0.938912$$ and the average sum of square residual error is $$e_{N}^{t} = 1.70109 \times 10^{ - 5}$$. Figure 2 achieves the sum average of residual errors when $$Pr = 6.2$$,$$ M = \delta_{1} = 1.0$$, $$f_{w} = 2.5$$, $$ S = - 0.5$$, $$ \delta_{2} = - 2.5$$, $$ \lambda = - 0.1$$, $$ n = 0.1$$, $$ Ec = 0.5$$,$$ We_{x} = We_{y} = 0.3$$, $$ K = R_{d} = N_{t} = N_{b} = 0.5$$, $$ m = 2.0$$, $$ \gamma = 1.0$$, and $$Le = 5$$. Table 1 demonstrates residual errors with similar parametric quantities as stated above for different approximation orders.

**Table 1:** Averaged residual squared errors taking into account the desired auxiliary number values.

$N$	$e_{N}^{f}$	$e_{N}^{{\text{g}}}$	$e_{N}^{\theta }$	$e_{N}^{\phi }$
4	$7.37908 \times 10^{ - 7}$	$5.15601 \times 10^{ - 7}$	$6.56997 \times 10^{ - 6}$	$9.12325 \times 10^{ - 6}$
10	$5.14596 \times 10^{ - 10}$	$4.11797 \times 10^{ - 10}$	$3.55419 \times 10^{ - 8}$	$9.2597 \times 10^{ - 8}$
20	$7.93954 \times 10^{ - 13}$	$2.38014 \times 10^{ - 14}$	$2.97324 \times 10^{ - 11}$	$8.930159 \times 10^{ - 11}$
30	$1.40898 \times 10^{ - 15}$	$8.6531 \times 10^{ - 17}$	$5.06211 \times 10^{ - 14}$	$1.50661 \times 10^{ - 13}$
40	$3.18976 \times 10^{ - 18}$	$4.58403 \times 10^{ - 20}$	$9.82761 \times 10^{ - 17}$	$2.77921 \times 10^{ - 16}$

## Results and discussion

This section demonstrates and discusses the outcomes of several leading parameters on the field of flow. We have organized our results by putting $Pr = 6.2, \;M = 1.0, \;\delta_{1} = 1.0, \;R_{d} = 0.5, \;K = 0.5, \;f_{w} = 2.5, \;S = - 0.5, \;\delta_{2} = - 2.5, \;\lambda = - 0.1, \;n = 0.1, \;Ec = 0.5, \;We_{x} = We_{y} = 0.3, \;N_{t} = N_{b} = 0.5, \;m = 2.0, \;\gamma = 1.0$, and $Le = 5$ except if specified otherwise. A comparison is exhibited in Table 2 in order to access the verification of the code of our study. The values of drag force for diverse values of $M$ taking the related parameters as $Pr = 1, \;n = 0, \;f_{w} = 0, \;\delta_{1} = 0,\;\delta_{2} = 0, \;\lambda = 0, \;K = 0, \;Ec = 0, \;S = 0, \;\gamma = 0, \;R_{d} = 0$ and $We_{x} = We_{y} = 0$. These values have been compared and showed excellent accord with Ref.^48^ and Ref.^51^.

**Table 2:** Comparison of drag force coefficient for distinct values of $M$ when $Pr = 1, \;n = 0, \;f_{w} = 0, \;\delta_{1} = 0,\;\delta_{2} = 0, \;\lambda = 0, \;K = 0, \;Ec = 0, \;S = 0,\; \gamma = 0, \;R_{d} = 0$ and $We_{x} = We_{y} = 0$.

$M$	Ref.^48^	Ref.^51^	Present outcomes
0	− 1.00000	− 1.00001	− 1.0000097
0.25	− 1.11803	− 1.11803	− 1.1180333
1	− 1.41421	− 1.41421	− 1.4142105
5	− 2.44949	− 2.44949	− 2.4494932
10	− 3.31662	− 3.31663	− 3.3166265
50	− 7.14142	− 7.14143	− 7.1414259
100	− 10.0499	− 10.0499	− 10.049894
500	− 22.383	− 22.383	− 22.383134
1,000	− 31.6386	− 31.6386	− 31.638602

### Impact of porous material parameter *K*

**Figure 3:**
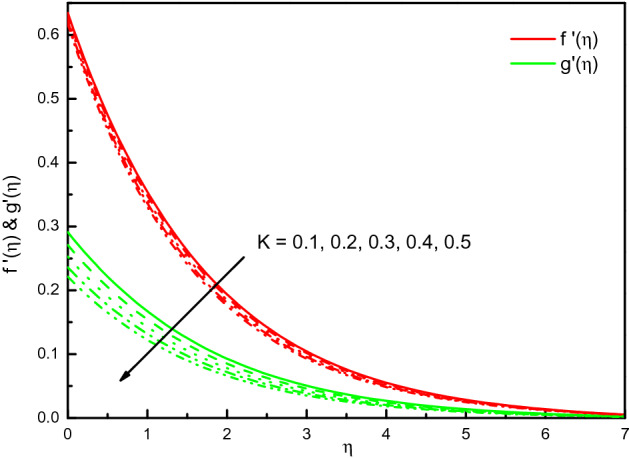
Graph of $f^{\prime}(\eta )$ and ${\text{g}}^{\prime}(\eta )$ vs. $K$.

**Figure 4:**
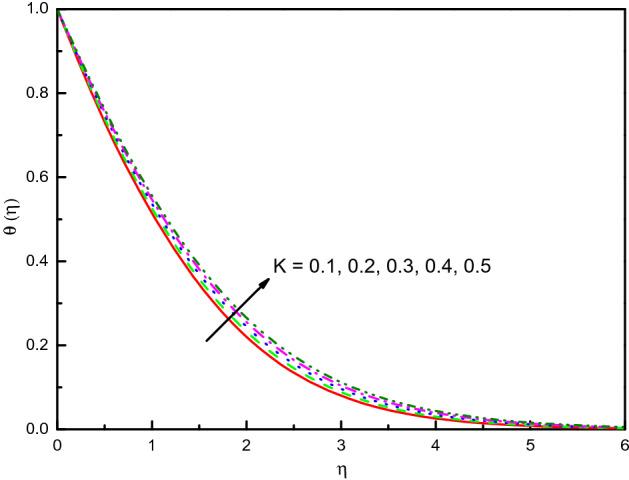
Graph of $\theta (\eta )$ vs. $K$.

**Figure 5:**
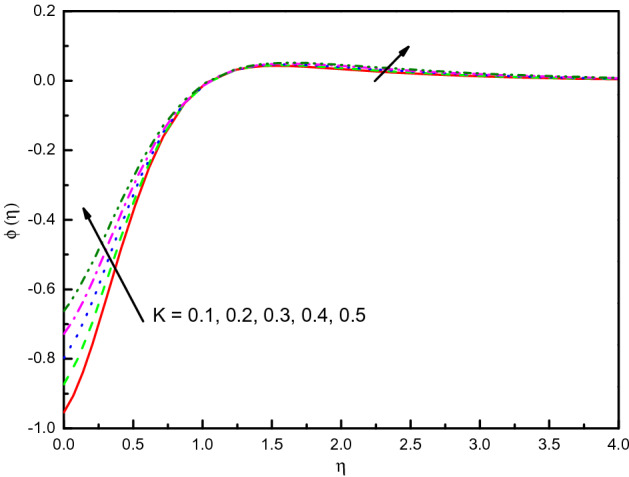
Graph of $\phi (\eta )$ vs. $K$.

Figure 3 reveals the velocities profile for different values of a porous material parameter $K$. It is found that the $x$ and $y$-axes velocities reduce with $K$, and $f^{\prime}(\eta )$ existence on the upper side. Thus, the thickness of the momentum's boundary-layer is reduced, and less volume from the nanofluid is fluxed. The cause behind the physical phenomenon is the pores of a porous material that declines the velocities. The effects are clear and distinct close to the surface. Pictographic proofs also reveal that nanofluid has a high velocity in $x$-direction $f^{\prime}(\eta )$ relative to the $y$-velocity ${\text{g}}^{\prime}(\eta )$. The profile of temperature $\theta (\eta )$ as we displayed in Fig. 4 depicts that the temperature of the nanofluid raises because of the escalating behavior of $K$. Within $1.0 \le \eta \le 4.0$ (not precisely specified), the influence is very obvious and significant. The curve converges smoothly to zero outside the area described above, namely $\eta > 4.0$. Figure 5 elaborates the concentration distribution $\phi (\eta )$ for growing the value of $K$. In the interior range $0.0 \le \eta \le 1.5$ (not precisely specified) the influence is distinguished but for $\eta > 1.5$, the curve is stable and asymptotically inclining to zero. This figure manifests that the nanofluid concentration is diminished more rapidly than that for the case without porous material. The reduced number of Sherwood $Sh_{x}$ should be noted is reduced for the mounting style of $K$.

### Impact of suction (blowing) *f*_*w*_

**Figure 6:**
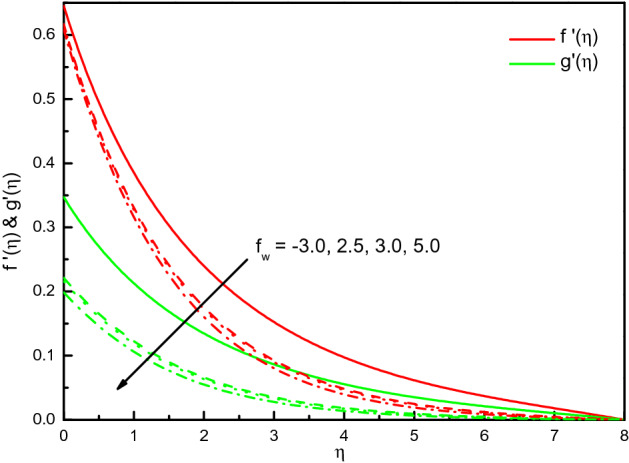
Graph of $f^{\prime}(\eta )$ and ${\text{g}}^{\prime}(\eta )$ vs. $f_{w}$.

**Figure 7:**
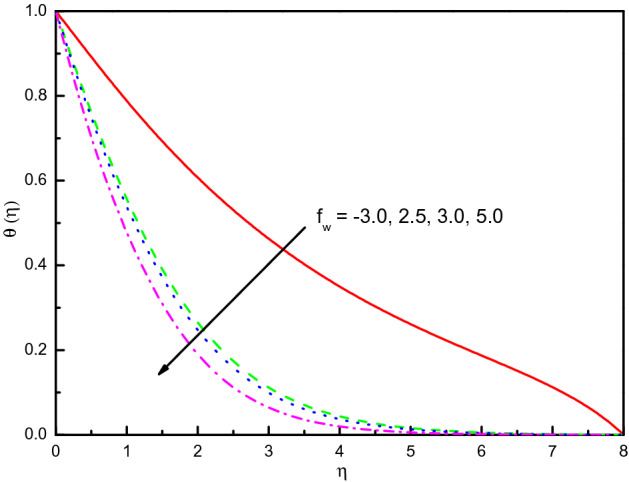
Graph of $\theta (\eta )$ vs. $f_{w}$.

**Figure 8:**
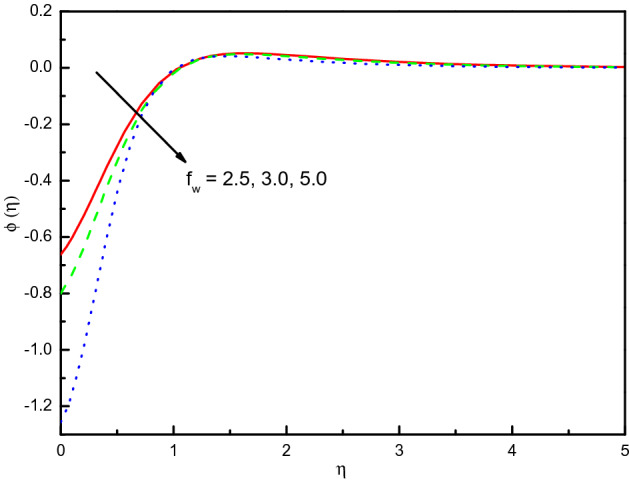
Graph of $\phi (\eta )$ vs. $f_{w}$.

Figure 6 offers the velocities profile for distinct values of a suction (blowing) parameter *f*_*w*_. It is remarked that the $$x$$ and $$y$$-directions flow velocities diminish with *f*_*w*_, and $$f^{\prime}(\eta )$$ existence on the top part. Thus, the thickness of the corresponding boundary-layer is reduced too, and the blowing $$(f_{w} < 0)$$ is a higher velocity than the suction $$(f_{w} > 0)$$. The cause behind the physical phenomenon is the pores of a porous material that declines the velocities. The influences are strong and distinct close to the surface especially in $${\text{g}}^{\prime}(\eta )$$. The profile of temperature $$\theta \left( \eta \right)$$ as exhibited in Fig. 7 addresses the temperature of the nanofluid diminishes cause of the intensifying behavior of *f*_*w*_. Within $$1.0 \le \eta \le 5.0$$ (not precisely specified), the impact is very noticeable and significant. The curve converges smoothly to zero outside the area described above, namely $$\eta > 5$$. It obvious that the blowing effect (at $$f_{w} = - 0.3) $$ is not asymptote in the established boundary conditions of the problem, because doesn't meet the tolerance of computations and there may be no flow at this value experimentally. Figure 8 explains the reduction of concentration gradients $$\phi (\eta )$$ for rising the value of $$f_{w}$$. Inside the range $$0.0 \le \eta \le 4.0$$ (not precisely specified) the influence is illustrious but for $$\eta > 4.0$$, the curve is stable and asymptotically predisposing to zero. This figure establishes that the nanofluid concentration is raised than that of without suction (blowing). The rate of mass transfer that should be observed is raised for the escalating modality of $$f_{w}$$.

### Impact of power-law index *n*

**Figure 9:**
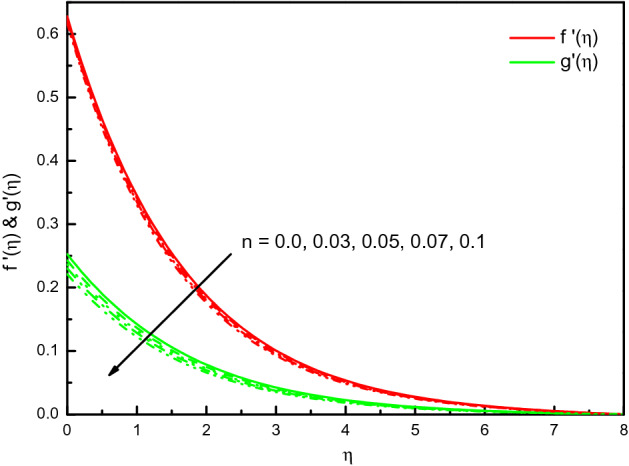
Graph of $f^{\prime}(\eta )$ and ${\text{g}}^{\prime}(\eta )$ vs. $n$.

**Figure 10:**
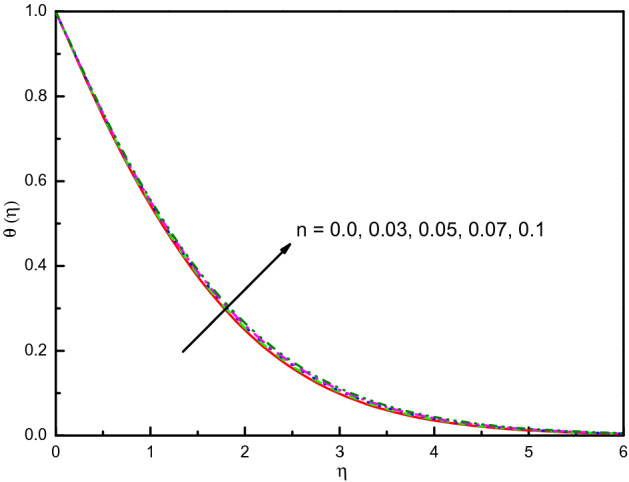
Graph of $\theta (\eta )$ vs. $n$.

**Figure 11:**
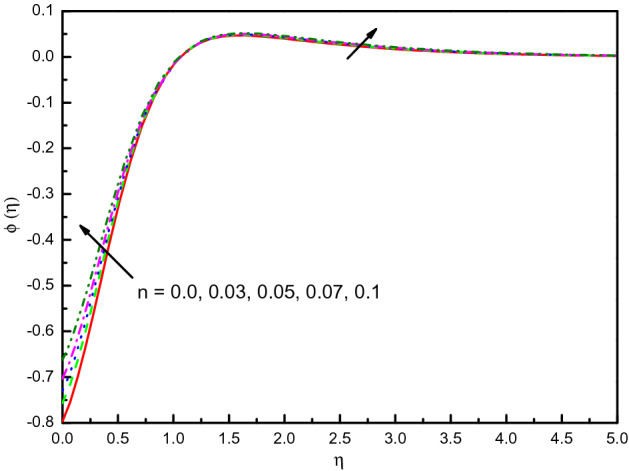
Graph of $\phi (\eta )$ vs. $n$.

Figure 9 illustrates the influence of $n$ on $f^{\prime}(\eta )$ and ${\text{g}}^{\prime}(\eta )$. Likewise, the non-Newtonian factor n amplification implicates high viscous properties in the nanofluid, and therefore reduces the impact. The results are transparent and distinct near to the wall. In graphical evidence, Newtonian nanofluid also acquires a high velocity compared to non-Newtonian nanofluid. This reduces the drag force's effectiveness on the surface by the Newtonian characteristic of the fluid. The temperature establishes to overabundant in Fig. 10 with the values of increment in $n$. In fact, the numerical findings support the important effects of nanofluid thermal properties on the consistency index $n$. Figure 11 indicates the concentration improved outlines and the consistent boundary-layer thickness. The rate of mass transfer is diminished with $n$ rising values.

### Impact of slip velocity ${\varvec{\delta}}_{1}$

**Figure 12:**
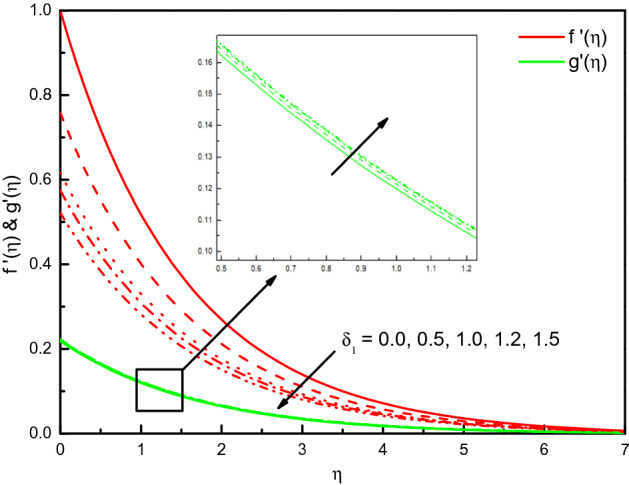
Graph of $f^{\prime}(\eta )$ and ${\text{g}}^{\prime}(\eta )$ vs. $\delta_{1}$.

Figure 12 elucidates that $f^{\prime}(\eta )$ is declined with the upsurge of $\delta_{1}$ parameter whilst, ${\text{g}}^{\prime}(\eta )$ is raised with $\delta_{1}$. As $\delta_{1}$ parameter is specified as $\sqrt {a\alpha } N_{1}$, an upsurge in the parameter $\delta_{1}$ will affect in a rise in thermal diffusion $\alpha$ and hence the thermal conductivity. We can see that thermal diffusion is nothing but the measurement of resistance to nanofluid movement especially in the $x$-velocity but the effect on ${\text{g}}^{\prime}(\eta )$ not clear relative to $f^{\prime}(\eta )$. Therefore, $\delta_{1}$ positive effect will certainly slow the nanofluid dominant velocity. The impact near to the surface is dominant, however further from the surface, it is diminished. Interestingly, the velocity of the Newtonian nanofluid in the boundary-layer region is altitude compared with others. Due to the resistivity force non-Newtonian nanofluid will be stronger because of the greater viscosity of it. The result is a higher velocity of the Newtonian nanofluids.

**Figure 13:**
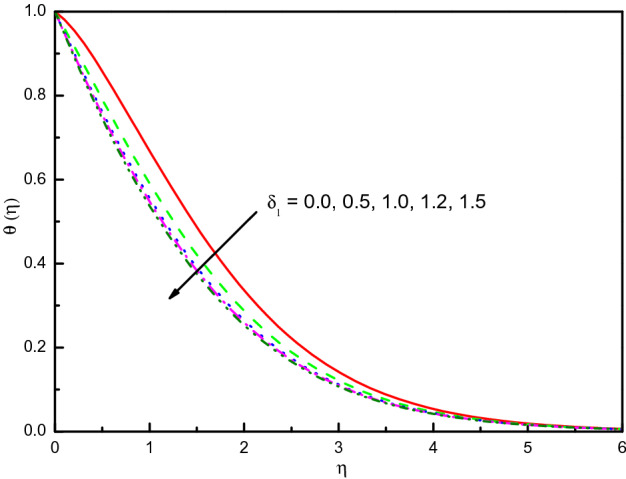
Graph of $\theta (\eta )$ vs. $\delta_{1}$.

**Figure 14:**
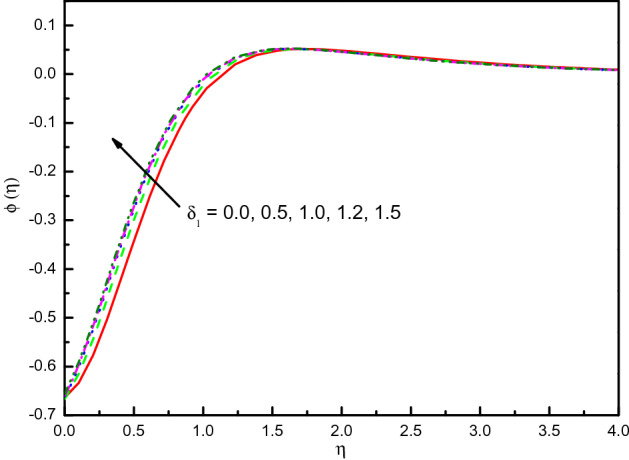
Graph of $\phi (\eta )$ vs. $\delta_{1}$.

Figure 13 reveals that the profile of temperature is diminished into the boundary layer result in the positive impact of $\delta_{1}$, and by the condition $\eta \to \infty$, the temperature concurs fluently. Physically, it can be clarified that the temperature is decreased because the parameter $\delta_{1}$ looks to be a desirable consideration for increasing thermal diffusion. It is decided that the rate of heat transport can be increased by $\delta_{1}$. The concentration outline $\phi (\eta )$ is magnified with the increment values of $\delta_{1}$ as shown in Fig. 14. In the area $0.0 \le \eta \le 2.0$ (not precisely specified) the influence is eminent but in the area after $\eta = 2.0$, the curve is approximately stable and tends to zero. It is obvious from this figure that $\phi (\eta )$ of a nanofluid without slip velocity is less in comparison with $\delta_{1}$ existence. Also, this gives us the expectation that $Sh_{x}$ shrinkages due to the mounting behavior of $\delta_{1}$.

### Impact of thermal radiation ${\varvec{R}}_{{\varvec{d}}}$, heat source ${\varvec{S}}$, and Eckert number ${\varvec{Ec}}$

**Figure 15:**
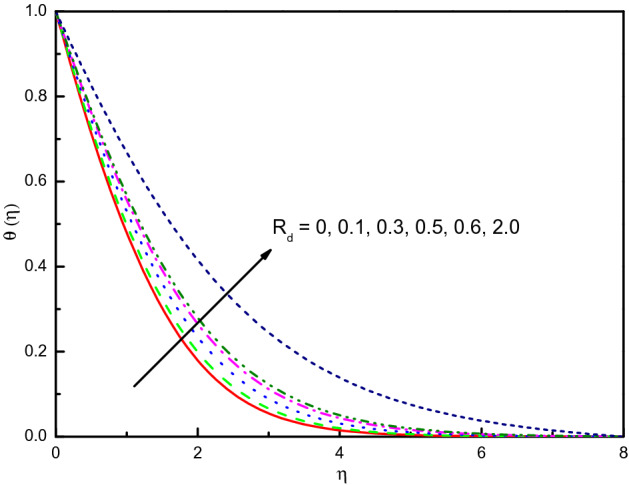
Graph of $\theta (\eta )$ vs. $R_{d}$.

**Figure 16:**
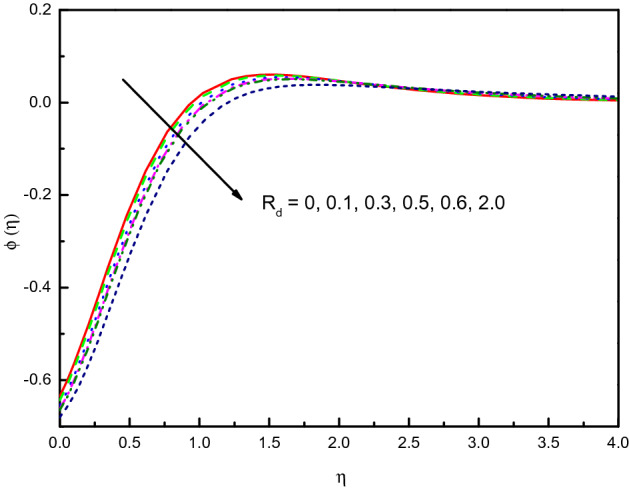
Graph of $\phi (\eta )$ vs. $R_{d}$.

Figure 15 discloses that the temperature for the nanofluid is raised due to the influence of thermal radiation parameter $R_{d}$. The impact within the region $1.0 \le \eta \le 4.0$ (approximately) is very significant and clear yet the curvature after that converges asymptotically to zero. This upsurges the thickening of the thermal boundary layer. The parameter $R_{d}$, i.e. $R_{d} = \frac{{4\sigma^{*} T_{\infty }^{3} }}{{3k^{*} k}}$ allows us to obtain the physical evidence that the nanofluid thermal conductivity weakens immediately $R_{d}$ rises because the frictional heat is decreased in the flow scheme. It is remarkable to remember that being extremely viscous in kind, hyperbolic tangent nanofluids produce more heat than Newtonian nanofluids. Figure 16 discloses that the concentration $\phi (\eta )$ diminishes in response to the non-negative values of $R_{d}$ when $R_{d} > 0$. The nanofluid has a less concentration profile with $R_{d}$ compared in the situation without $R_{d}$, this can be traced to a promisingly effective concentration within the area $0.0 \le \eta \le 2.5$ (not precisely specified).

**Figure 17:**
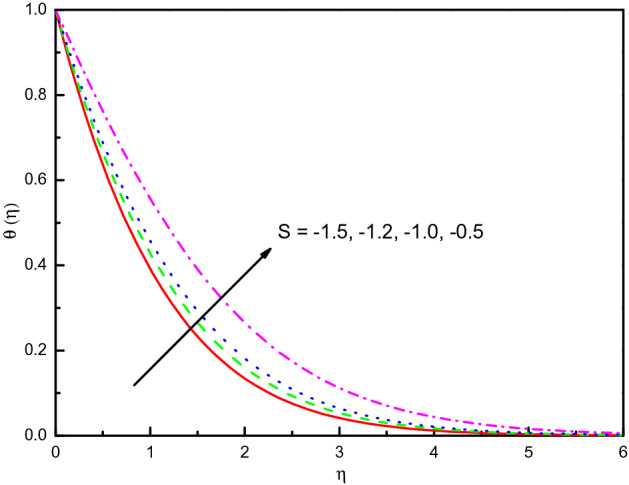
Graph of $\theta (\eta )$ vs. $S$.

**Figure 18:**
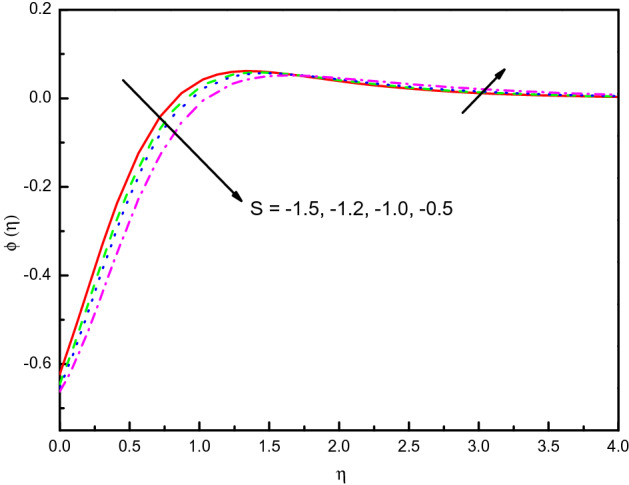
Graph of $\phi (\eta )$ vs. $S$.

**Figure 19:**
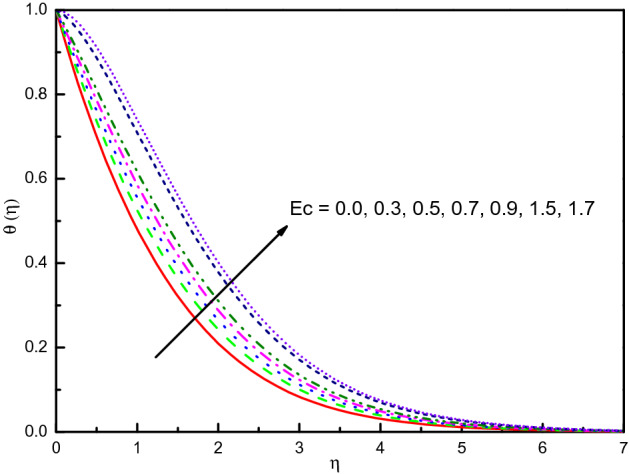
Graph of $\theta (\eta )$ vs. $Ec$.

**Figure 20:**
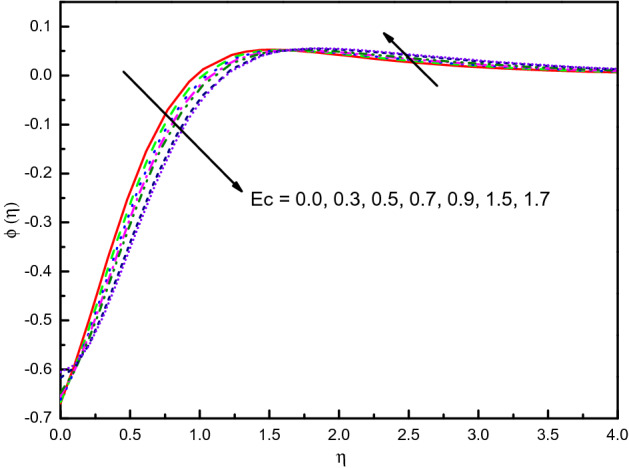
Graph of $\phi (\eta )$ vs. $Ec$.

The outcome of $S$ on temperature and concentration outlines has been disclosed in Figs. 17 and 18, respectively. It is noticed that temperature tends to rise and concentration inclines to diminish and the influence is protuberant within section $0.0 \le \eta \le 2.5$. The curve then attains the boundary condition numerically and graphically. Correspondingly, the temperature and concentration distribution predominate much as we discussed in the absorption case. The same impact can be remarked in the case of growing values of $Ec$ on the $\theta (\eta )$ and $\phi (\eta )$ which is displayed Figs. 19 and 20. But the higher $Ec$ values are more clearly temperature than concentration, it is notable that after the value $\eta > 1.5$, the effect of the concentration is reversed. From Table 3, we see that $\left| { - \theta^{\prime}(0)} \right|$ declines for both $R_{d}$, $S$, and $Ec$.

**Table 3:** Variations of $\left| { - \theta ^{\prime}(0)} \right|$ with $Ec, \;S$ and $R_{d}$ when $Pr = M = \gamma = m = K = 1,\; f_{w} = 2.5, \;\delta_{1} = 1,\;\delta_{2} = - 2.5, \;\lambda = 0.1, \;n = 0.5, \;We_{x} = We_{y} = 0.3, \;N_{t} = N_{b} = 0.1$, and $Le = 5$.

$Ec$	$S$	$R_{d}$				
		0.3	0.5	1.0	1.5	2.0
0.0	− 0.5	0.5832	0.0253	0.4548	0.4027	0.3658
	− 0.3	0.4491	0.0253	0.3523	0.3136	0.2865
	0.0	0.1109	0.1131	0.0253	0.1183	0.1195
	0.5	0.0330	0.1991	0.8399	5.4013	2.0063
0.1	− 0.5	0.5786	0.5318	0.4520	0.4005	0.3640
	− 0.3	0.4444	0.4092	0.3494	0.3113	0.2847
	0.0	0.1059	0.1088	0.1134	0.1159	0.1175
	0.5	0.0381	0.1951	0.8375	1.1382	2.1485
0.5	− 0.5	0.5603	0.5163	0.4407	0.3916	0.3566
	− 0.3	0.4256	0.3933	0.3379	0.3023	0.2772
	0.0	0.0856	0.0918	0.1012	0.1065	0.1098
	0.5	0.0585	0.1788	0.8280	1.1629	4.2189

### Impact of chemical reaction ${\varvec{\gamma}}$ and its order ${\varvec{m}}$

**Figure 21:**
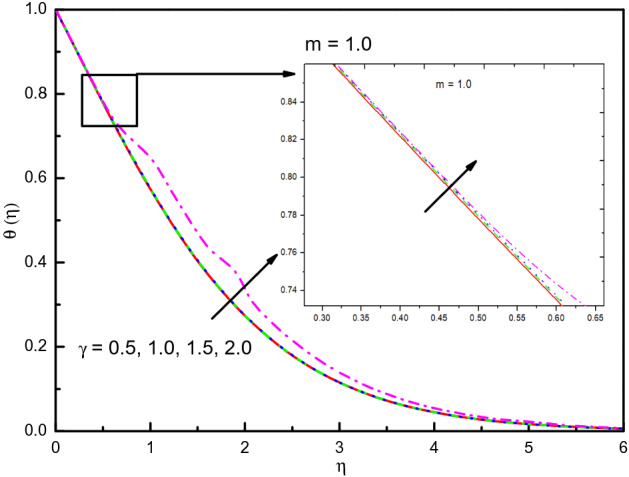
Graph of $\theta (\eta )$ vs. $\gamma$.

**Figure 22:**
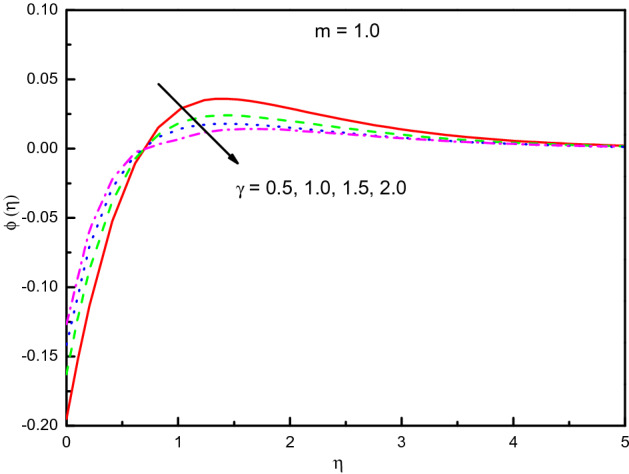
Graph of $\phi (\eta )$ vs. $\gamma$.

Figure 21 shows that in feedback to the positive method of parameter $\gamma$ in $\gamma > 0$ (destructive reactions), the temperature in the 1st-order of reaction (i.e., $m = 1.0$) increases. In the area of $0.5 \le \eta \le 4.0$ (not precisely specified), the temperature is efficient auspiciously. Figure 22 reveals that the concentration is raised with the $\gamma$ is the destructive case in the area $0.0 \le \eta \le 1.0$ approximately, after that the impact is reversed. Furthermore, the well-known formula between the parameter of the rate of chemical reaction and volume concentration is specified as $\gamma \propto (\phi )^{m}$, where $m$ is the reaction order. The relationship offers that if $\gamma$ increases, the concentration goes high in $\eta > 1.0$, after that is reversed until $\eta = 4$, then it converges to zero. But when we look at the destructive reaction $(\gamma > 0)$, the result is totally reverse and some disturbance in the flow happened.

**Figure 23:**
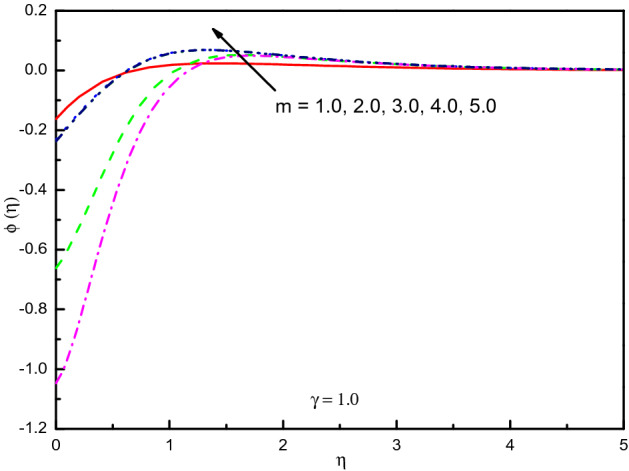
Graph of $\phi (\eta )$ vs. $m$ (destructive reaction).

**Figure 24:**
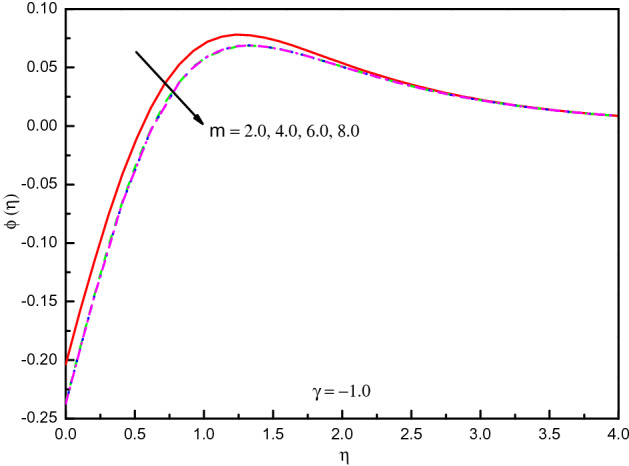
Graph of $\phi (\eta )$ vs. $m$ (generative reaction).

It is seen from Fig. 23 that the concentration of destructive $(\gamma = 1.0)$ case upsurges in the most area of $\eta$ but it is mentionable the impact $m$ varies between the odd and even order when the order of the reaction increased. The impact is significantly eminent here on concentration. However, Fig. 24 for $\gamma = - 1.0$ shows the opposite result. For $\gamma < 0.0$, In the area $0.0 \le \eta \le 2.5$ (approximately) the curve purely rises, but then the curve gradually reduces and converges to zero asymptotically. The concentration distribution is not so eminent in the situation of a generative reaction with $m$ after $m > 4$. Table 4 confirms the graphical outcomes, whilst the rate of mass transport is diminished with $\gamma$ in the case of a destructive reaction, this impact raises the rate with $m$ and it varies with $m > 4$. In this case, it is obvious that $Ec$ diminished the rate of mass transport.

**Table 4:** Variations of $\left| { - \phi ^{\prime}(0)} \right|$ with $Ec, \;m$ and $\gamma$ when $Pr = M = \delta_{1} = R_{d} = K = 1, \;f_{w} = 2.5, \;S = - 0.5,\; \delta_{2} = - 2.5, \;\lambda = 0.1, \;n = 0.1, \;We_{x} = We_{y} = 0.3, \;N_{t} = N_{b} = 0.5$, and $Le = 5$.

$Ec$	$m$	$\gamma$				
		0.1	0.5	1.0	1.5	2.0
0.0	1.0	0.4468	0.4395	0.4366	0.4350	0.4339
	3.0	0.4486	0.4463	0.4453	0.4448	0.4443
	5.0	0.4481	0.4465	0.4459	0.4456	0.4453
	7.0	0.4475	0.4464	0.4459	0.4456	0.4455
0.5	1.0	0.4183	0.4111	0.4082	0.4067	0.4057
	3.0	0.4201	0.4178	0.4169	0.4163	0.4159
	5.0	0.4196	0.4181	0.4175	0.4171	0.4169
	7.0	0.4191	0.4179	0.4175	0.4172	0.4170
1.0	1.0	0.3898	0.3827	0.3798	0.3783	0.3774
	3.0	0.3916	0.3893	0.3884	0.3879	0.3874
	5.0	0.3911	0.3896	0.3890	0.3887	0.3884
	7.0	0.3906	0.3895	0.3890	0.3888	0.3886

## Conclusions

Due to its extensive applications in cancer medications, drug delivery, polymers, and optical fiber, the new features of thermal ray and higher-order reactions in the flow behavior of non-Newtonian nanofluids are very interesting. The influence of thermal ray, and $m{\text{th}}$-order chemical reaction on hyperbolic tangent nanofluid flow in a porous material with heat generation (absorption), and Joule heating has been discussed by utilizing the OHAM. Some significant findings are listed on the basis of the entire investigation:

- The velocities distributions decline, when the parameter of porous material is escalated. The same behavior with $\delta_{1}$, $f_{w}$, and $n$, except the impact of $\delta_{1}$ on the ${\text{g}}^{\prime}(\eta )$.
- Temperature improves with increasing values of $K$, $n$, and the generative reaction case. But the reverse state occurs with $f_{w}$, $\delta_{1}$, and the destructive reaction.
- Concentration diminishes with $R_{d}$, $Ec$, and the destructive reaction, but the reversed effect observes with $n$ and $m$ (destructive state). The increment is significant with $K$ and $\delta_{1}$, whereas $f_{w}$ and $S$ offer reducing phenomenon near the boundary-layer.
- Nusselt number reduces in the existence of $R_{d}$, $Ec$ and $S$ parameters, whilst the contrary state happens in the situation of $f_{w}$.
- Sherwood number reinforces with $Ec$, and $R_{d}$ are increased. Nonetheless, the opposite influence is featured with $K$ and $m$ in the case of destructive reaction.

## Abbreviations

$$a,b$$: Constants (–)
$$B_{0}$$: Magnetic parameter
$$C$$: Concentration (mol/m^3^)
$$c_{p}$$: Specific heat
$C_{\infty }$: Free stream concentration (mol/m^3^)
$C_{w}$: Constant-wall concentration (mol/m^3^)
$$D_{B}$$: Brownian diffusion (m^2^/s)
$$D_{T}$$: Thermophoresis diffusion (m^2^/s)
$$Ec$$: Eckert number (–)
$f,{\text{g}}$: Dimensionless stream functions (–)
$f_{w}$: Suction/injection parameter (–)
$k$: Thermal conductivity (W/mK)
$K$: Porous medium permeability (–)
$K_{1}$: Porous medium parameter (–)
$$k_{c}$$: Chemical reaction (–)
$k^{*}$: Mean-absorption factor
$$Le$$: Lewis number (–)
$$M$$: Magnetic parameter (–)
$m$: Order of reactions (–)
$n$: Power-law index (–)
$N_{b}$: Brownian motion parameter (–)
$$N_{t}$$: Thermophoresis parameter (–)
$N_{1} ,\; N_{2}$: Coefficients of slip velocity (–)
$Q$: Heat source (sink) (W/m^2^ K)
$q_{r}$: Radiative heat flux (W/m^2^)
$$R_{d}$$: Thermal ray (–)
$$S$$: Heat source (–)
$$Pr$$: Prandtl number (–)
$$T$$: Temperature (K)
$T_{w}$: Constant-wall temperature (K)
$T_{\infty }$: Free-stream temperature (K)
$u,v,w$: Velocity components (ms^−^^1^)
$We_{x} ,\;We_{y}$: Numbers of Weissenberg (–)
$w_{0}$: Suction (injection) parameter (–)
$$x,y,z$$: Space coordinates (m)

## Greek symbols

$$\alpha = \frac{k}{{\rho c_{p} }}$$: Thermal diffusivity $$({\text{J/m}}^{3} \cdot {\text{K}})$$
$\delta_{1} ,\; \delta_{2}$: Velocity slip parameters
$$\gamma$$: Chemical reaction (–)
$$\eta$$: Similarity parameter (–)
$$\theta$$: Dimensionless temperature
$\lambda$: Constant parameter of sheet (–)
$\mu$: Dynamic viscosity (Pa s)
$$\nu$$: Kinematic viscosity (m^2^/s)
$$\rho$$: Density (kg/m^3^)
$(\rho c_{p} )_{f}$: Heat capacity of the base fluid ${\text{(J/kg}} \cdot {\text{K}})$
$(\rho c_{p} )_{s}$: Heat capacity of a nanoparticle ${\text{(J/kg}} \cdot {\text{K}})$
$\sigma$: The electrical conductivity
$\sigma^{*}$: Constant of Stefan–Boltzmann
$\tau = \frac{{ (\rho c_{p} )_{s} }}{{(\rho c_{p} )_{f} }}$: Ratio between the heat capacities (–)
$$\phi$$: Dimensionless concentration (–)
$${\Gamma }$$: Williamson parameter (–)

## Subscripts

$\infty$: Free stream condition
$$f$$: Base fluid
$$s$$: Solid nanoparticle
$$w$$: Wall

## Abbreviations

3-D: Three-dimensions
OHAM: Optimal homotopy analytical method
MHD: Magnetohydrodynamics
MRI: Magnetic resonance imaging
PDEs: Partial differential equations
ODEs: Ordinary differential equations
